# A Comparative Study of Localized Corrosion and Stress Corrosion Cracking of 13Cr Martensitic Stainless Steel Using Acoustic Emission and X-ray Computed Tomography

**DOI:** 10.3390/ma12162569

**Published:** 2019-08-12

**Authors:** Kaige Wu, Kaita Ito, Ippei Shinozaki, Pornthep Chivavibul, Manabu Enoki

**Affiliations:** 1 Department of Materials Engineering, The University of Tokyo, 7-3-1 Hongo, Bunkyo-ku, Tokyo 113-8656, Japan; 2 Research and Services Division of Materials Data and Integrated System, National Institute for Materials Science, 1-1 Namiki, Tsukuba, Ibaraki 305-0044, Japan; 3 Materials Department, Research Laboratory, IHI Corporation, 1 Shin-Nakahara-Cho, Isogo-ku, Yokohama 235-8501, Japan

**Keywords:** martensitic stainless steel, pitting, stress corrosion cracking, plastic deformation, acoustic emission, X-ray CT

## Abstract

An accurate evaluation of stress corrosion cracking (SCC) in 13Cr martensitic stainless steel (MSS) is still missing due to the lack of an in-situ insight into the process evolution and full characterization of the corrosion morphology. In this work, two main regimes involved in the SCC progression, including localized corrosion and cracking, were comparatively studied using in-situ acoustic emission (AE) monitoring and three-dimensional (3D) X-ray computed tomography (XCT) scanning. The stress corrosion tests were conducted with u-bent smooth specimens subjected to a single droplet of 1 μL 1% neutral NaCl solution. Localized corrosion and cracking evolution were controlled in tempered and quenched steel specimens, respectively. From XCT scanning, localized corrosion was featured by an irregular corrosion pit with deposited corrosion products containing cracks. The single dominant SCC crack was observed to initiate from corrosion pit and propagate with a 3D tortuous and discontinuous morphology. AE signals were detected in both cases. Correlated with in-situ observations and clustering analysis, source identification of AE signals was proposed. AE signals during localized corrosion were assessed to be mainly from cracking within the deposited corrosion products. Comparatively, hydrogen-bubble evolution, plastic deformation, and crack-branches coalescence were proposed as the AE sources of cracking evolution.

## 1. Introduction

Due to its good mechanical properties and reasonable corrosion resistance, 13Cr martensitic stainless steel (MSS) has been widely used as a structural material in many fields like civil engineering, nuclear power plants, and the oil & gas industry. However, it can undergo stress corrosion cracking (SCC) when exposed to the combination of stress and corrosive environment, and especially the chloride-containing environment [1]. In many applications, localized corrosion serves as the precursor to SCC [2]. Compared with the rapid cracking regime, the long-term localized corrosion can largely determine the overall life of an exposed component. Therefore, surveillance of the early localized corrosion and subsequent cracking evolution are of equal importance to SCC monitoring. In practical application, to reduce the risk of premature failures of high-strength structural components in hostile fields, establishment of in-situ monitoring methodologies for the SCC is always required.

Acoustic Emission (AE) method is a promising nondestructive testing (NDT) method with high sensitivity to various corrosion processes [3]. The AE method has been used to study steel corrosion like localized corrosion [4,5,6,7,8,9,10,11,12,13] and SCC [14,15,16,17,18,19,20,21,22,23,24,25,26]. Numerous experimental works have deepened our understanding of the damages during corrosion processes based on the recorded AE signals. First, AE activities during localized corrosion have been assigned with different damage processes, including instantaneous stress changes on the metal surface [4], rupture of pit cover [5,6], breakdown of the passivating film [7], corrosion potential fluctuations [8], and hydrogen bubble evolution [5,6,9,10,11,12,13]. Comparatively, the different damages process during SCC evolution, including hydrogen bubble evolution [14,15,16,25], plastic deformation of the advancing crack tip [17,18], cracking within the corrosion products [14,19,20,21], falling off of the surface grain [14,19,21], and propagation of the primary crack [15,16,17,18,19,22,23,24,25,26] have been proposed in literature as the possible mechanisms for generation of AE signals. On the other hand, some AE signal-derived secondary parameters have been introduced as a discriminant factor to estimate the morphological evolution of pitting corrosion [6], or to monitor the SCC mode [18]. Furthermore, the detected AE signal has been combined with electrochemical noise to distinguish the different damage stages with a predominant electrochemical or mechanical contribution to crack propagation depending on the SCC evolution [15].

Despite extensive work and the advances that have been made, many challenges still exist in evaluating and understanding AE behavior during SCC. First of all, most source mechanisms during SCC proposed in literature are not undisputed since the interpretation of AE data is mainly based on post-mortem microstructural analysis. The primary reason for this challenge lies in the fact that most accelerating corrosion tests, mostly derived from ASTM G129 [27], ASTM G36 [28], or NACE TM0177 [29] are basically conducted in bulk solutions relevant to complete immersion conditions. This greatly increases the difficulty in observing the surface of immersed specimen in-situ. Although some AE signals during SCC have been interestingly correlated with the concurrent electrochemical noise [14,15,26], limited in-situ observational data seems to make their conclusions not so visualized. Meanwhile, some SCC testing in hot magnesium chloride solution [15] can introduce additional uncertainty in extracting the AE events from the artificial noises. Moreover, the localized corrosion as the precursor to cracking has not gained enough attentions compared with the crack propagation. In many reports [24,26,30,31], early localized corrosion during SCC was often determined to be undetected or detected with rare AE events, which obviously conflict the AE detectability of localized corrosion verified by many other works [4,5,6,7,8,9,10,11,12,13]. This may be attributed to the principle of accelerated corrosion testing in most laboratory study for shortening experimental time periods. By this consideration, the time to crack initiation is always intended to be reduced either by tailoring the specimen’s microstructure to be more susceptible or by applying more aggressive environment. This can prematurely trigger the condition for pit-to-crack transition when the pit is still in its infancy, corresponding to the phenomenon of “delay time” (i.e., a time delay for pit growth to reach a critical size required for AE detection) [5,6,9,11,12,13] and therefore unmeasured by AE method [30,31]. This can lead to lost sight of some secondary AE source mechanisms like the cracking of deposited corrosion products and hydrogen bubble evolution in developed pits that should have gained attention in practical applications.

On the other hand, any corrosion evolution is a process not just evolving on the specimen surface, but developing spatially in three dimensions (3D). Therefore, two-dimensional (2D) techniques cannot characterize all the local features with the inner corrosion morphology. The recently-developed nondestructive 3D technique of X-ray computed tomography (XCT), whether synchrotron-based or laboratory setups, has been applied to study the localized corrosion, intergranular corrosion, and SCC of stainless steels and aluminium alloys [32,33,34]. The application of tomographic 2D images and rendered 3D views has enabled remarkable progress in characterizing and understanding the evolution of corrosion damages. A combination of in-situ and ex-situ experiments has produced a quantitative evaluation of the morphology of localized corrosion, as well as the pit-to-crack transition in steels [32]. A counterintuitive fact was uncovered from 3D tomographic imaging that the crack is not necessarily initiated from the pit base but tends to primarily develop from the pit mouth [33]. Moreover, some transgranular cracks that seem to be discontinuous from the surface observation were confirmed by XCT to be actually continuous inside the specimen by demonstrating the angular extending of some secondary micro-cracks emanated from the main propagation direction of a main crack [34]. XCT has gained wide popularity with many interesting results in corrosion studies. However, study on the localized corrosion and SCC in MSSs was rarely reported.

In this work, motivated by the steel corrosion under a thin aqueous layer relevant to atmospheric corrosion [19,22,30,31,35], a stress corrosion test under a single droplet of neutral NaCl solution was improved in the u-bent SUS420J2 MSS specimen to facilitate AE monitoring with in-situ optical microscopy observations. A direct support to AE data analysis was expected to be provided based on in-situ observational data of the corrosion process. On the other hand, the evolution of localized corrosion and cracking development was controlled by the specimen preparation with different heat treatment procedures (i.e., tempered vs. as-quenched). XCT was used to demonstrate the corrosion morphologies of both cases. Meanwhile, the AE behaviors of both cases were comparatively studied. From an AE clustering analysis, in-situ observations, correlative post-observations, and source identification of AE signals was discussed.

## 2. Materials and Methods

### 2.1. Materials and Specimen Preparations

A commercial 13Cr MSS of SUS420J2 (provided by Kusayama Unique Special Steel, Kawasaki, Japan.) was used in this study. The chemical composition of the steel is given in Table 1. Two rolled flat bars of 22 mm in thickness were austenitized at 950 °C for 2 h, followed by quenching in argon gas. Then, one quenched steel bar was additionally tempered at 500 °C for 20 h, followed by air cooling. The other bar was used as as-quenched specimen. After heat treatment, cylindrical tensile specimens with a gauge length of 30 mm and a diameter of 6 mm were produced from the two steel bars for characterization of the mechanical properties of both steels in accordance with Japan Industrial Standard (JIS) G0567 [36] at room temperature. The tensile tests were conducted using a universal testing machine (Instron 5982, Norwood, MA, USA) with a loading capacity of 100 kN. The mechanical properties of both steels are summarized in Table 2, where *E* is the modulus of elasticity, *σ*_0.2_ is the 0.2% proof stress, UTS is the ultimate tensile stress, EL, RA, and HV represent the elongation, reduction in area, and Vickers hardness. Two steels with distinct properties were intended to have differing corrosion behavior. Two kinds of corrosion specimens with identical dimensions of 170.0 mm (L) × 6.3 mm (W) × 1.5 mm (T) were produced for corrosion testing from the tempered bar (marked as specimen A) and quenched bar (marked as specimen B). All corrosion specimens were polished down to 1 μm diamond suspension before corrosion testing.

### 2.2. Microstructure Characterization and Precipitation Identification

Specimens were observed using optical microscopy (OM) and field-emission scanning electron microscopy (FE-SEM, JSM7000F, JEOL Ltd., Tokyo, Japan) equipped with energy-dispersive spectroscopy (EDX) after being chemically etched in Villella’s reagent (5cc hydrochloric acid + 2g Picric acid +100cc Ethyl alcohol) for 5 s. Moreover, X-ray diffraction (XRD) measurements were conducted using a 9-kW diffractometer (Cu Kα radiation source; Rigaku SmartLab, Rigaku Corp., Tokyo, Japan) after the specimens were wet-polished down to 0.05 μm colloidal silica suspension. The 2θ-scan range was set as 40° to 90° with a step size of 0.01° and a scan speed of 2°/min to analyze the main structure of each specimen. To analyze the detailed information of the precipitates [37], a scan ranging from 47° to 53° of 2θ was further performed with a 0.01° step size and a 0.1°/min scan speed.

### 2.3. SCC Testing and AE Measurements

**Figure 1:**
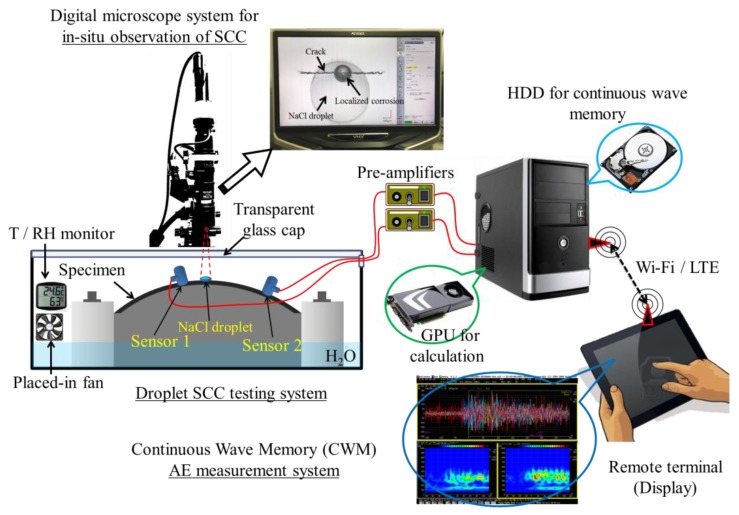
Schematic of the modified stress corrosion cracking (SCC) test system incorporated with an in-situ digital microscope and acoustic emission (AE) measurement systems. Online version in color.

Figure 1 shows the experimental setup. The specimen was bent and fixed in an experimental jig with a curvature radius of 125 mm leading to a strain of 0.6% at the apex of the specimen surface, which is approximated by the equation [38]: ε = *t*/2*R*, where *t* is the specimen thickness and *R* is the radius of the bend. A single droplet of 1 μL 1.0 mass% neutral NaCl solution was dropped in the center area of the specimen surface using a microliter syringe. The experimental apparatus was placed in a thermostatic bath (298 K, 99%–100% humidity) with pure water. The bath was covered with a transparent glass cap to allow in-situ observation using a digital microscope (VHX-5000, Keyence Corp., Osaka, Japan). Considering the constant deformation of the u-bending specimen and localized corrosion region under the droplet, the overall stress and strain of specimen during the testing was believed to be uniform and constant compared to the initial bending state. But as the corrosion proceeded, the local stress and strain near the corrosion region were estimated to increase and drive the crack nucleation. Simultaneously, continuous AE measurement was performed using a custom AE acquisition system developed in our group, continuous wave memory (CWM) [39]. Two high-sensitivity R-CAST sensor systems with a resonant frequency of 200 kHz (M204A, Fuji Ceramics Corp., Fujinomiya, Japan, 55 dB gain) were positioned 10 mm and 30 mm away from the droplet site to record the AE signals. A threshold of 29.7 dB and a 100 kHz high-pass filter (HPF) were used as thresholds to isolate AE events from the continuously recorded waveform. Then the target signals were extracted by filtering out the noise outside the corrosion region using the source location method. K-means clustering algorithm was used to classify the extracted AE data of both cases [31]. The optimum number *k* of clustering analysis was determined by using the average silhouette coefficient [30,31]. After different AE clusters were recognized, the distributions of AE data were compared with the in-situ observational data of the corrosion process over the time evolution. The source mechanisms of detected AE signals were proposed by both considerations of the AE features and corresponding observations of the corrosion process.

### 2.4. X-ray CT and SEM-EBSD Measurements

After corrosion testing, the specimens were cut to an appropriate length of 20 mm to fit the Scanning electron microscopy (SEM) chamber and ultrasonically cleaned with ethanol at room temperature to remove the surface corrosion products. The surfaces of specimens were first inspected with SEM. Next, the spatial features of the corrosion morphologies were obtained by a 3D scanning using lab-based X-ray computed tomography (XCT) system (ZEISS Xradia 520 Versa, Carl Zeiss XRM, Pleasanton, CA, USA). A full scan of 360° rotation of the specimen was conducted at a tube voltage of 140 kV and a power of 10 W with a scanning period of 8.5 h. A resolution of 1.0 μm/voxel was achieved with a field of view (FoV) width of 2 mm. After scanning, the acquired projection images were imported into the software ORS Dragonfly Pro (Object Research System, Montreal, Canada) to produce a 3D reconstruction of the specimen’s volume. Moreover, to investigate the local feature around the crack tip, electron backscatter diffraction (EBSD) measurement was performed after a fine polishing of the crack area with a 0.05 μm colloidal silica suspension for 5 h. EBSD data were collected using a field-emission SEM (JSM70001, JEOL, Tokyo, Japan) equipped with a EBSD detector. A 15-kV acceleration voltage was selected with a step size of 0.25 μm for EBSD scanning. EBSD data were analyzed using OIM (EDAX-TSL) v7 software (EDAX Inc., Mahwah, NJ, USA). The Kernel Average Misorientation (KAM) analysis was used to assess the local plastic deformation around the crack tip [30]. KAM during EBSD analysis is a qualitative quantification of the average local misorientation around a measurement point with respect to all of its nearest neighbor points by definition in the perimeter of the kernel. Deformed locals due to high dislocation density correspond to high KAM intensity. KAM can be used to evaluate the localized deformation on the well-prepared specimen surface containing high-density dislocation. For the as-quenched specimen B containing high-density dislocation with itself, KAM map was calculated using third nearest-neighbor EBSD points and a maximum of 6° to track the localized plasticity along the crack tip. Meanwhile, the cross-sectional morphology of localized corrosion was observed using SEM with a serial sectioning of the specimen.

## 3. Results

### 3.1. Microstructure and Precipitates

**Figure 2:**
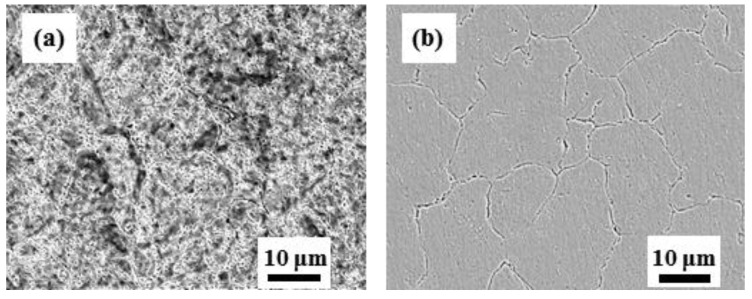
Microstructures of chemically etched specimens (Viella’s reagent for 5 s): (**a**) specimen A; (**b**) specimen B.

**Figure 3:**
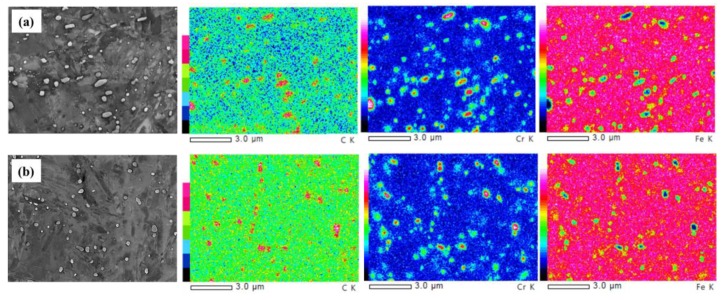
Scanning electron microscopy-energy-dispersive spectroscopy (SEM-EDX) map showing carbon and chromium enrichment and iron depletion on precipitates. (**a**) specimen A; (**b**) specimen B. Online version in color.

**Figure 4:**
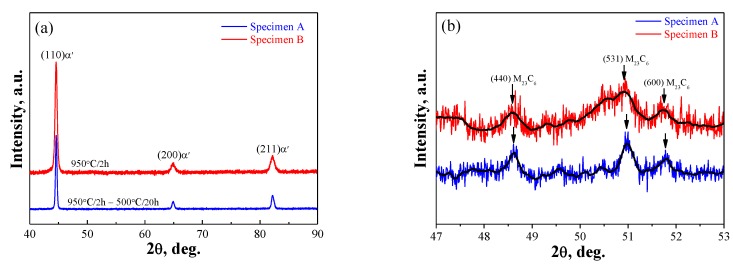
X-ray diffraction (XRD) patterns of the two steel specimens: (**a**) diffraction angles from 40° to 90° and (**b**) detailed information between diffraction angles from 47° to 53°. Online version in color.

Figure 2 shows a general view of the microstructure of tempered and quenched specimens. Figure 3 shows the localized microstructure with EDX mapping, indicating that carbides rich in Cr and C decorate the hierarchic lath structured martensitic matrix which agrees well with the other work [40]. Prior austenite grain boundaries appear more visibly in quenched microstructure B with smaller precipitates up to 0.5 μm; while in tempered specimen A, some nano-sized precipitates (<0.1 μm) are also observed in addition to the submicro-sized carbides with sizes up to 0.9 μm. All precipitates exhibit similar circular or elongated shape. X-ray diffraction (XRD) spectra of specimens A and B are shown in Figure 4, indicating the (110), (200), and (211) planes of martensite (α′) phase only. Some weak peaks within 47° to 53° of both steel are determined to be 48.60°, 50.90°, 51.80°, which could indicate the (440), (531), and (600) planes of Cr-rich M_23_C_6_ carbides. It is consistent with the results of TEM observation of the same steel tempered at 500° [37] and 550° [41]. Therefore, the submicro-sized precipitates in both steels seem to be undissolved Cr-rich M_23_C_6_ carbides, and some additional nano-sized Cr-rich M_23_C_6_ carbides were formed during the tempering process for specimen A.

### 3.2. Corrosion Processes

Three parallel experimentations of both steels were conducted in the same condition with similar results. Only one typical data for each case was reported in this paper. 

**Figure 5:**
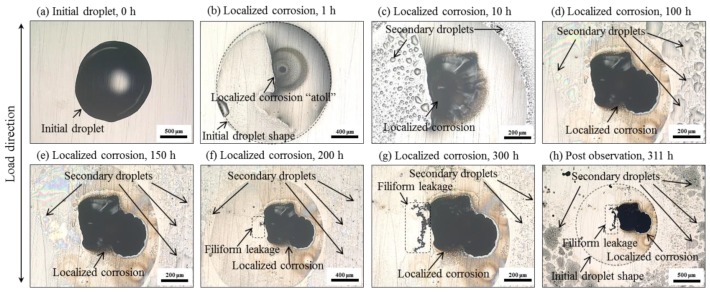
Localized corrosion in the u-bent specimen A with 311 h’ exposure to a single droplet of 1 μL neutral 1% NaCl solution in 99% relative humidity at room temperature: (**a**–**g**) in-situ observation; (**h**) post-observation.

Figure 5 shows the in-situ observations of the corrosion process in specimen A. Early in the beginning of the experiment, localized corrosion began as the droplet shrank. A typical feature of corrosion “atoll” was gradually visualized as the formation of an annular, ring-like surface stains under the droplet (Figure 5a–c). This corrosion atoll is attributed to the atmospheric corrosion under local NaCl attack developed with spatial separation of the anodic and cathodic areas [30,31,42]. The deposited corrosion products were yellowish initially and then became dark, spreading over the entire corrosion atoll (Figure 5d–g). During this process, many secondary droplets formed adjacent to the original droplet. As corrosion proceeded, some secondary droplets coalesced to larger droplets (Figure 5c–e). In the late stage (Figure 5f–h), these secondary droplets gradually disappeared away from the localized corrosion site. This phenomenon was also captured by another researcher [42] who thought that these secondary droplets originated from both the initial droplet and moisture in the atmosphere and may serve as the cathodic area. Anodic location in the central corrosion site with high acidity, low potential, and high Cl^−^ enhanced the metal dissolution within the hydrogen reduction [30]. Moreover, a filiform leakage of corrosion solution (Figure 5f–h), similar to the observation in [42], from the localized corrosion was captured, but no AE event was recorded along with this phenomenon. Finally, no crack initiation was triggered, and only localized corrosion deposited with corrosion products was observed.

**Figure 6:**
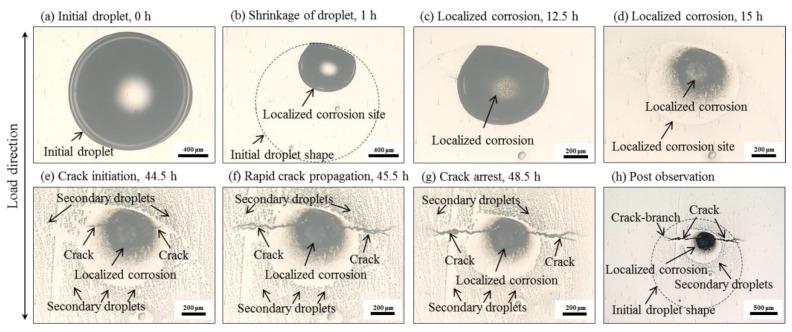
A single crack evolution in the u-bent specimen B exposed to a single droplet of 1 μL neutral 1% NaCl solution in 99% relative humidity at room temperature until the crack arrest in 48.5 h: (**a**–**g**) in-situ observation; (**h**) post-observation.

Figure 6 shows the in-situ observations of the corrosion process in specimen B. As expected, initiation and propagation of SCC was observed with relatively shorter exposure than specimen A. Generally, the crack initiated from the early localized corrosion. In similarity to the case of specimen A, the early stage of corrosion evolution in specimen B could be mentioned as localized corrosion which was featured by a corrosion atoll with localized attack (Figure 6a–d). Thereafter, the crack was observed to develop from both sides of the localized corrosion normal to the u-bend load direction. The crack grew with a low rate in the beginning (Figure 6e), then transitioned to a high growth rate with the increasing crack length (Figure 6f–g). Many secondary droplets spreading along the perimeter of the localized corrosion area, which is similar to the case of specimen A, were also observed. A phenomenon of crack-branch coalescence during crack propagation was captured and would be discussed in detail with AE data. The test was finally stopped with the occurrence of crack arrest, which was believed to be controlled by the electrochemical, mechanical, and geometric factors [43], but was not included in the primary concern of this work.

### 3.3. Corrosion Morphologies

**Figure 7:**
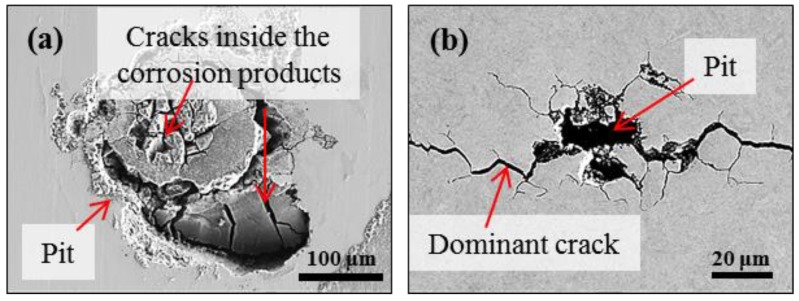
Scanning electron microscopy (SEM) images showing the surface morphologies of corroded specimens A (**a**) and B (**b**).

The local morphology of corrosion areas of two cases after testing was compared from the surface observations. In specimen A, some cracks can be recognized within some corrosion products deposited inside an irregular pit, as shown in Figure 7a. Figure 7b shows the local features related to the crack initiation in specimen B. A single dominant crack is observed to be from a corrosion pit. Some small secondary cracks were also observed to have emanated from the main propagation direction of the main crack.

**Figure 8:**
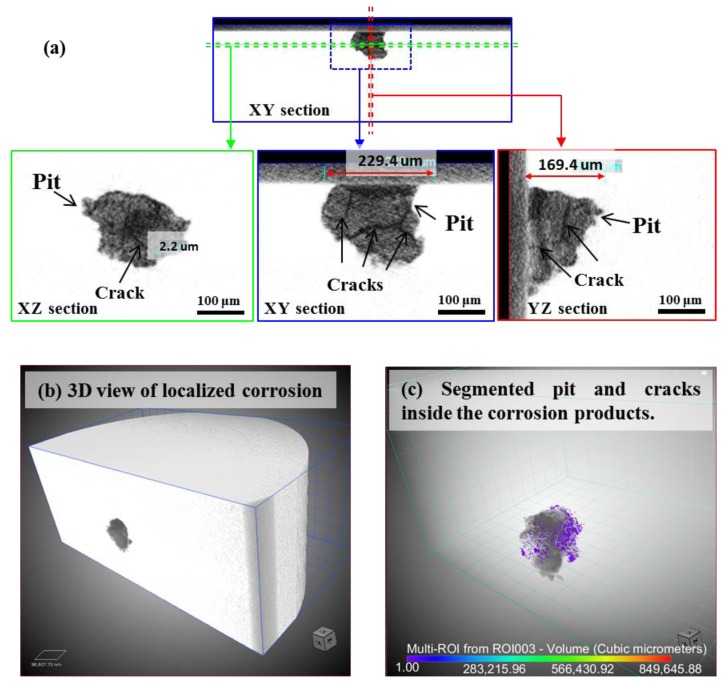
XCT data of localized corrosion of specimen A: (**a**) Virtual slices in different sections; (**b**) 3D tomogram; (**c**) Segmented pit and cracks inside the corrosion products with transparent volume of material. Here, the color is based on the volume. Online version in color.

Figure 8 shows the reconstructed slices and volume rendering images from the XCT data of localized corrosion in specimen A. From the unique observation of inner morphology in different sections, the localized corrosion featured by an irregular pit with deposited corrosion products containing cracks as small as the micron scale is clearly confirmed. No SCC was observed from the bottom or the shoulder of pit thanks to the full scan of the corrosion region, indicating that damages during the corrosion process of specimen A were mainly the pitting corrosion and precipitation of corrosion products with cracking. A 3D view and a segmentation of the localized corrosion were also tried. Despite some uncertainty existing in segmenting the small crack inside the corrosion products, the spatial geometry of the corrosion pit inside a MSS was first provided. The main merit of this 3D visualization of a real pit is probably the potential in triggering a data-driven modeling of the crack initiation in relation to the consideration of real pit geometry, rather than the introduction of a fictive semi-sphere or ellipse as the initial defect [33,34].

**Figure 9:**
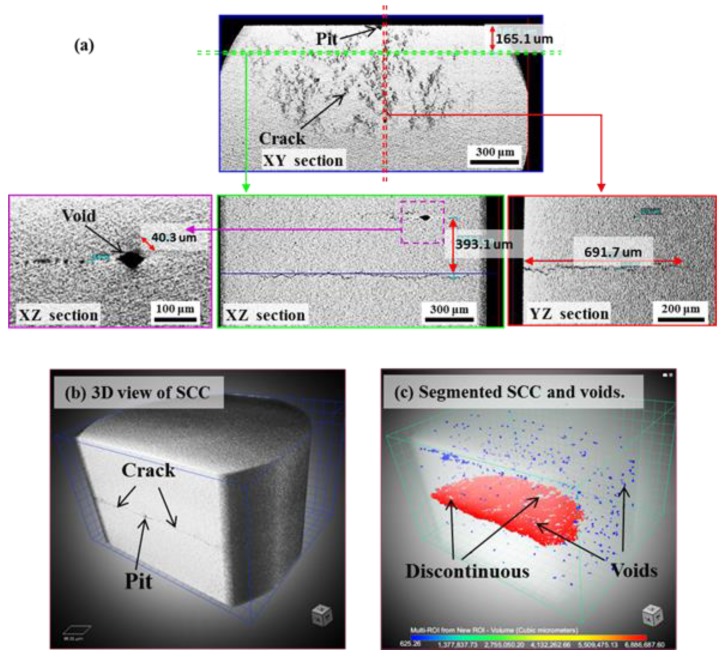
X-ray computed tomography (XCT) data of SCC of specimen B: (**a**) Virtual slices in different sections; (**b**) 3D tomogram; (**c**) Segmented crack and voids with transparent volume of materials. Here, the color is based on the volume. Online version in color.

Figure 9 shows the XCT data of SCC in specimen B. First, it allows a direct illustration the relative spatial position of the corrosion pit and crack. Combined with surface observation in Figure 7b, it clearly supports that the crack initiated from the corrosion pit was as similar as the mostly reported cases [2,15,16,19,29,30,31,32,40]. A zig-zag morphology could be related to the crack propagation from the X-Z and Y-Z sections. Some voids up to dozens of micrometers are dispersedly visible in material. The void formation is probably associated with the localized shear deformation and distortion during the quenching process. The caused residual stress around these voids may accelerate the crack initiation and propagation. Additionally, the main crack is seemingly continuous at large as a single crack, which is similar to the claim in the SCC of austenitic stainless steel [34]. However, the crack evolution inside the present material is more complex as tortuous morphology from a 3D view than the zig-zag morphology from 2D tomographic images or SEM surface observation. First, some voids appearing at the crack plane locally separated the crack to be discontinuous. Secondly, compared with the reported 3D crack in Al-7Si-1Mg alloy [44], the real morphology of SCC in MSS is believed to be largely affected by an uneven growth from a localized competition among the crack fronts because of a stress shielding effect inside the heterogeneous material. A detailed interpretation of the fracture mechanisms around the crack-tip fields requires time-lapse imaging and in-situ AE monitoring of the crack growth, which will be reported in another paper.

Table 3 summarized the experimental results of both steels. In summary, localized corrosion with no SCC initiation was observed in tempered specimen A until 311 h’ exposure. Localized corrosion is featured by an irregular corrosion pit where corrosion products deposited with cracks; while in quenched specimen B, crack initiation and propagation were observed and finally stopped with crack arrest in 48.5 h. The crack was observed to be initiated from a corrosion pit and propagated with a 3D tortuous and discontinuous path, rather than the *pseudo-continuous* from 2D characterization.

### 3.4. AE Results

**Figure 10:**
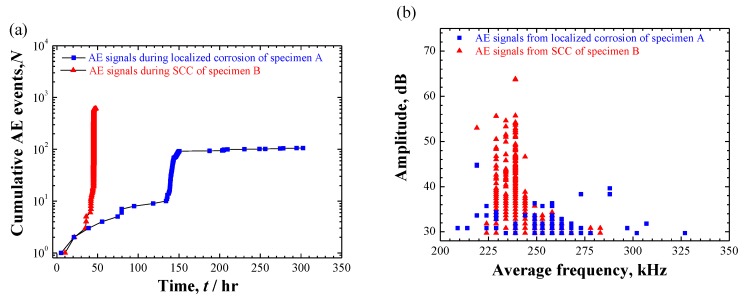
Comparison of the AE signals extracted from the corrosion processes of specimens A and B: (**a**) Cumulative AE events over time; (**b**) Cross-plot of amplitude to average frequency. Online version in color.

As summarized in Table 3, 106 and 610 AE signals were extracted from the localized corrosion and crack evolution, respectively. Figure 10 shows a comparison of extracted AE signals over the time evolution and in cross-plot. Overall, all AE signals distribute in a resemble frequency range between 200–350 kHz. However, crack evolution in specimen B produced more AE activity with higher amplitude-level than the localized corrosion of specimen A. K-means clustering analysis was applied to the AE data and the results of AE clustering analysis, as well as the source identification of two cases will be discussed separately.

#### 3.4.1. AE Signals during Localized Corrosion in Specimen A

**Figure 11:**
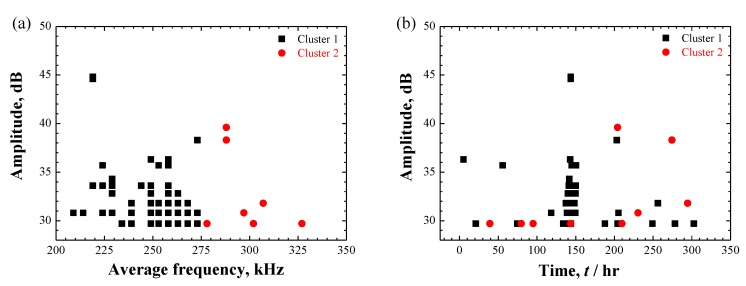
(**a**) The result of AE clustering analysis with two clusters; (**b**) Evolution of cluster 1 and 2 during localized corrosion in specimen A. Online version in color.

**Figure 12:**
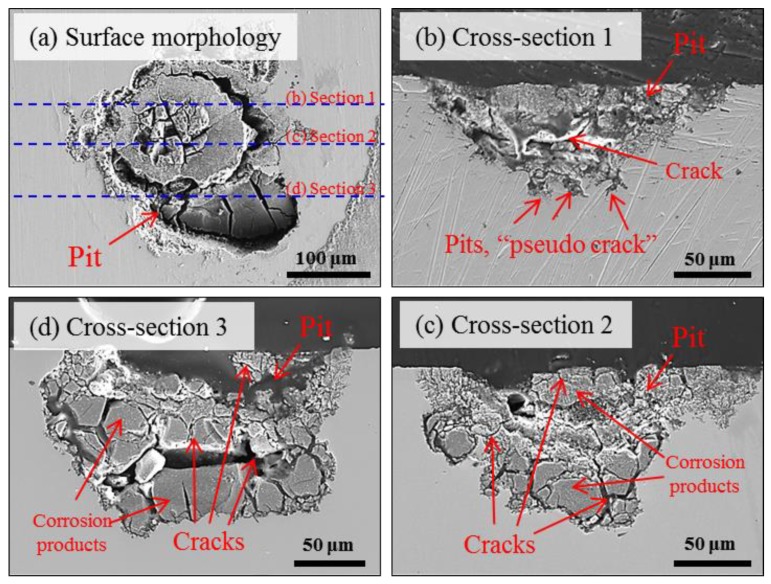
SEM images via serial sectioning of the localized corrosion area in specimen A: (**a**) surface morphology and (**b**–**d**) Observations of cross-sections 1–3. Online version in color.

As presented in Figure 11a, two clusters with different frequency level were obtained, i.e., low-frequency cluster 1 and high-frequency cluster 2. Figure 11b shows the time evolution of two AE clusters. A weak tendency could be recognized, i.e., low-frequency cluster 1 was first detected and high-frequency cluster 2 began to appear in the middle and later periods of the corrosion duration. To assist in interpreting AE signals, a further cross-sectional observation of the localized corrosion was implemented. Figure 12 shows three sectional morphologies of the selected sections. More clearly, corrosion products containing cracks are observed inside the irregular pit. The cracks seem to cross through the corrosion products in a disorderly fashion (Figure 8). Some micro-pits are also shown at the base of the macro-pit. They were marked as *pseudo-crack* since no high-amplitude signals were recorded during the localized corrosion compared to the SCC in specimen B. The mechanism accounting for localized corrosion has been deeply studied in relation to the chloride ion attack. By considering the transport of ionic species, the metal dissolution followed by hydrolysis of metal ions could maintain an active dissolution interface which drives the pit growth [35,42,45,46]. Along with this process, the involved hydrogen-bubble evolution was mostly proposed in literature as the source for AE signals with higher-frequency or lower-strength than cracking-signals recorded during localized corrosion [5,6,7] and SCC [24,25]. Therefore, the high-frequency cluster 2 is attributed to the hydrogen-bubble evolution despite being without direct-observations.

Cracks within the corrosion products are believed to be generated with the precipitation and accumulation of corrosion products that were frequently quoted as a secondary AE source during localized corrosion and SCC [8,14,19,20,21]. The low-frequency cluster 1 was accordingly associated with the cracking within the corrosion products. Moreover, hydrogen-bubble evolution is generally thought to occur after the dissolution of metal and formation of occluded locals with low-pH condition [5,6,9,13,30,45,46]. The distribution of cluster-2 in middle and later periods of the corrosion process, as well as lagging behind cluster 1, may logically support the source identification of AE signals during localized corrosion.

Moreover, an obvious increase in the AE population was observed around 140–150 h, as shown in Figure 10a and Figure 11b. This phenomenon was also confirmed in repeated trials. However, from the in-situ surface observations (Figure 5), no exact change could be determined around this point because of the covered corrosion products. Considering the most signals from cracking within the corrosion products, this phenomenon may reflect a transition associated with the accumulation of corrosion products. A reliable interpretation requires an interrupted experiment in future works.

#### 3.4.2. AE Signals during SCC in Specimen B

**Figure 13:**
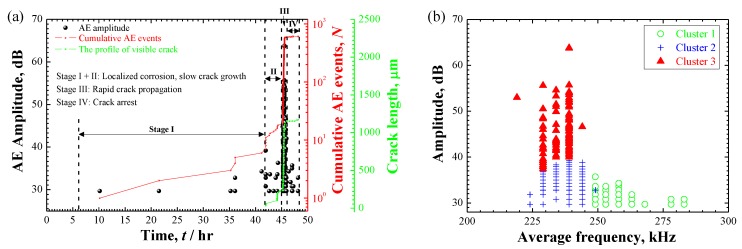
(**a**) The AE activity of amplitude and cumulative AE events and the profile of visible crack over time evolution and (**b**) the result of clustering analysis showing three AE clusters with different AE features during SCC evolution in specimen B. Online version in color.

Figure 13a shows the AE results with the profile of visible cracks from the in-situ observation of specimen B. By the appearance of corrosion evolution and AE data, four stages were identified. Stage I and II were observed to be localized corrosion and slow crack growth with relatively moderate AE events. However, the detailed separation between the localized corrosion and crack initiation was difficult to obtain because the whole crack-profile was masked by the corrosion products. In stage III, the crack shifted to a high growth rate with profuse AE signals. Finally, crack arrest occurred in stage IV. According to a detailed discussion of the initiation and propagation of SCC in another work [30], the slow and fast crack growth were supposed to be corresponding with the initiation and propagation of SCC which were predominantly controlled with active path corrosion and hydrogen-assisted cracking mechanisms, respectively. The distinction in number of AE signals of stage II and III may reflect the initiation-to-propagation transition during SCC progression. Figure 13b shows the results of clustering analysis and three clusters with different AE features were recognized, i.e., cluster 1 with low-amplitude and high-frequency, cluster 2 and 3 with resembled frequency components but with a distinct strength in amplitude. By a direct correlation with the hydrogen-bubble evolution captured in [31], high-frequency cluster 1 was attributed to be the hydrogen-bubble evolution.

**Figure 14:**
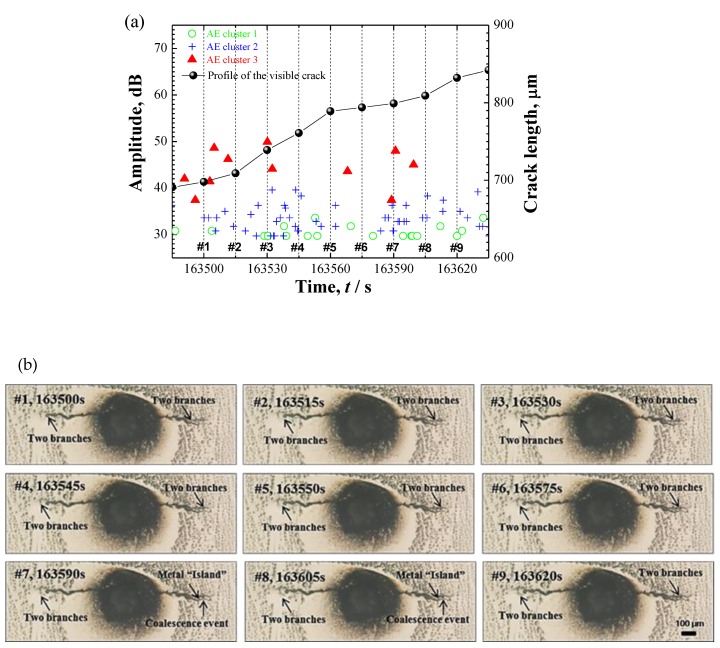
(**a**) The evolution of three AE clusters during the selected duration; (**b**) A phenomenon of crack-branch coalescence during crack propagation in specimen B. Marked numbers are corresponding to Figure 14a. Online version in color.

**Figure 15:**
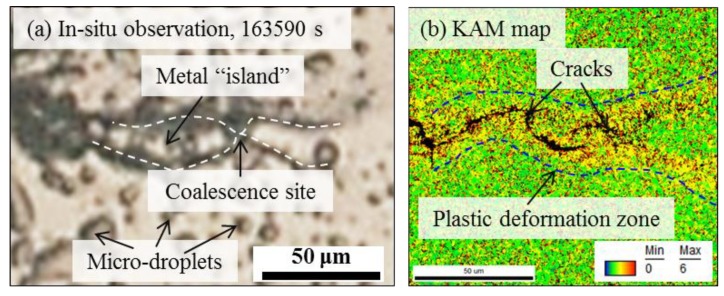
(**a**) In-situ observation of one coalescence event at the advancing crack tip; (**b**) KAM map of the corresponding area calculated using third nearest-neighbor EBSD points and a maximum of 6° showing the localized plastic deformation zone. Online version in color.

To interpret clusters 2 and 3, the distribution of AE data and in-situ observations during a selected period were compared as shown in Figure 14. Figure 14a shows the evolution of AE data and Figure 14b shows the successive capture of crack propagation. A phenomenon of crack-branches coalescence was monitored, as previously shown in another work [31], i.e., the crack propagated to both directions with the extending and coalescence of two crack branches at each crack tip. Images 6–8 highlight one coalescence event with the formation of a “*metal island”*. Interestingly, the high-amplitude cluster 3 was always concurrent with the occurrence of crack-branch coalescence. Therefore, the crack-branch coalescence may account for the AE cluster 3. Cluster 2 during the crack propagation may be from the plastic deformation process along with cracking growth. To support it, EBSD analysis of an advancing crack tip is shown in Figure 15. First, the coalescence site of the crack-branch and the metal island were well identified for the phenomenon of crack propagation. A plasticity zone along the crack path and tip was well confirmed with the high-intensity from the KAM map, indicating the local plastic deformation. As an important source for AE signals, plastic deformation during crack propagation is proposed to be the source mechanism of cluster 2 that is with resembling frequency components, but lower amplitude compared with the cluster 3 of cracking evolution. In a summary, the parameter of average-frequency separated the signal of hydrogen-bubble evolution (cluster 1); Cluster 2 and 3 having resembling frequency components but a different amplitude level were attributed to the plastic deformation and cracking propagation, respectively.

## 4. Discussion

### 4.1. Effect of Heat Treatment on the Corrosion Behavior of 13Cr MSSs

13Cr MSSs, based on a Fe-Cr-C ternary system, are usually thermally-treated to austenitic condition at elevated temperatures and then quenched to martensite state with body centered tetragonal crystal structure. Subsequently, as-quenched steels are often tailored to be with specific microstructure by tempering for a required combination of strength and ductility. For a 13Cr MSS with given chemical composition, the susceptibility to localized corrosion and SCC is largely dependent on its microstructure in relation to the residual stress due to austenite-to-martensite phase transformation during quenching treatment and Cr-rich secondary precipitation (mainly carbides) during tempering [37,40,41]. The matrix sites next to Cr-rich carbides have been confirmed to be a preferential site to localized corrosive attack [37]. Despite the disadvantage in precipitating Cr-rich secondary carbides (Figure 3 and Figure 4), tempering treatment at 500 °C is usually preferred as a standard procedure for 13Cr MSSs because of effective stress relaxation. In this work, localized corrosion as the early precursor stage to SCC was intentionally controlled in a 500 °C-tempered steel specimen. Initiation and propagation of SCC were intended to be accelerated in the specimen of as-quenched condition with high-residual stress and low toughness. From the results, the heat treatment-based corrosion behaviors were confirmed to be reliable to simulate two main regimes of SCC evolution, i.e., early precursor of localized corrosion and the later stage of crack evolution. Moreover, the improved stress corrosion testing relevant to droplet corrosion was proven to be a simple preparation, with high repeatability and effectiveness in accommodating the in-situ optical observation and AE monitoring experiments.

### 4.2. Relationship between Corrosion Process and AE Behavior

AE method as an effective NDT method has long been used to study the SCC of stainless steel. Extensive works in literature can be found on the AE behavior during SCC with face-centered cubic structured austenitic-grade stainless steel. However, less is known to the case with MSSs. In this work, the AE behaviors with two typical corrosion processes were comparatively studied with improved droplet SCC test. First, the AE activity both in population and feature is largely determined by the corrosion processes. In details, localized corrosion produced relatively weaker signals over longer-term exposure than the accelerated initiation and propagation of SCC. Second, with 3D views of the internal information of corrosion morphologies by XCT, the corrosion products containing cracks were confirmed in the localized corrosion of tempered specimen-A only, indicating that the condition for pit-to-crack transition in quenched specimen-B had been met early when pit was at its smaller size (Figure 7, Figure 8 and Figure 9, Figure 12) before corrosion products deposited inside it. Considering the standard heat treatment of specimen-A, AE signals during localized corrosion are worth being paid more attentions despite being weak. Since long-term localized corrosion in early stage of SCC evolution largely determines the lifetime of an exposed structure and component. The distinction of AE features between localized corrosion in specimen A and cracking propagation in specimen B could be used for in-situ corrosion monitoring against SCC-induced premature failures.

Moreover, it is of great significance to separate the localized plastic deformation (Figure 14, Figure 15) from the cracking process using the amplitude-parameter. It was proposed that AE amplitude is fundamentally related to the length scale and velocity of the AE source [47]. As further demonstrated in [48], various AE sources could be related to approximate ranges of amplitude, indicating the potential of AE amplitude in discriminating the processes of dislocation motion during plastic deformation (approx. 30–40 dB at sensor) and cracking (approx. 40–80 dB at sensor). This rough criterion may support the classification of present AE data during cracking propagation (Figure 13, Figure 14). It is conventionally recognized that the hydrogen-assisted cracking mechanism in relation to the hydrogen embrittlement (HE) plays a dominant but not necessarily exclusive role in the SCC evolution of high-strength martensitic steels [1,30,40]. However, there is still a strong disagreement among researchers on the inherent nature of HE mechanism. The localized plasticity around the crack tip and the concurrent AE signals (cluster 2) seem to be inclined to support a role of hydrogen-enhanced localized plasticity (HELP) model in cracking process based on hydrogen-enhanced dislocation mobility for HE mechanisms [49,50]. Considering the amplitude-parameter is relatively independent on the selected threshold, the use of AE amplitude parameter would demonstrate another potential application in in-situ revealing the HE mechanism, which has some advantages over the traditional methods of microscopy.

### 4.3. Uncertainty on AE Classification and Source Identification

SCC evolution is a complex process involving various events depending on both spatial and temporal evolution. The small dimensions, short timescale, and dynamic interplay between microstructure and changing electrochemical variables complicate the development of a complete understanding of the process evolution and whole morphology. From this work, 3D XCT technique and in-situ AE method were employed to comparatively study the localized corrosion and cracking evolution during SCC of a 13Cr MSS. Some spatial and temporal features related to SCC evolution were uncovered. With AE clustering analysis, in-situ observation, and correlative analysis, source mechanisms for recorded AE data were proposed. The identification of AE sources seems to be plausible. However, it must be emphasized that some uncertainty in AE classification and source identification still exist for several reasons. First, the damage events during corrosion process are always featured by mixture of long-termed incubation and instantaneous occurrence under corrosive electrolyte. The microscopic characteristics collected by conventional optical observation, either post-mortem or in-situ, cannot be corresponded one-on-one with their AE responses that are on the order of microseconds. Although in this work an attempt of in-situ observation was performed to reduce the time gap between recorded AE signals and observed damage events, the conclusions were indeed drawn based on some assumptions and speculations. Second, there is no universal clustering strategy in data science which can guarantee a perfect recognition between different AE populations. The unsupervised K-means algorithm used in this work presented reasonability in AE classification and interpretation. However, further proof based on improved clustering algorithm like supervised-machine learning and deep learning should be explored in future works.

Concluding this section, it should hold an open view with respect to the statistical analysis of AE data and source identification of the SCC process because uncertainty can be caused by a number of factors. More experimental works including finer testing design and advanced analysis are necessary to get closer to the nature of corrosion evolution and the concomitant AE behavior.

## 5. Conclusions

To better understand the initiation and propagation of SCC in 13Cr MSSs, localized corrosion as precursor evolution to SCC was simulated in a tempered specimen and cracking evolution was accelerated in an as-quenched steel specimen of SUS420J2. A comparative study of localized corrosion and cracking evolution were conducted using AE and XCT methods. Some conclusions were drawn based on the present experimental work.

(1) The corrosion susceptibility of SUS420J2 seemed to largely depend on the microstructure in relation to the Cr-rich M_23_C_6_ carbides and residual stress due to phase transformation during quenching treatment. With an improved droplet SCC testing, localized corrosion as the early precursor stage to SCC was intentionally controlled in a 500 °C-tempered steel specimen-A. Initiation and propagation of SCC were intended to be accelerated in the specimen-B of as-quenched condition with high-residual stress and low toughness.
(2) Localized corrosion was featured by an irregular pit with deposited corrosion products containing cracks; while the SCC was observed to be initiated from a corrosion pit and propagated with a 3D tortuous and discontinuous morphology that might be segmented by some voids, rather than the *pseudo-continuous* from 2D characterization.
(3) AE signals were detected in both cases. In case of localized corrosion, two clusters with different frequency level were recognized. Low-frequency AE cluster was believed to be from the cracking within the deposited corrosion products; high-frequency cluster was proposed to be associated with hydrogen-bubble evolution. Contrastingly, three AE clusters were divided in the case of crack propagation. Except for the high-frequency hydrogen-bubble evolution, plastic deformation and cracking process seemingly could be separated by the use of AE amplitude-parameter. The distinction of AE features between localized corrosion and cracking propagation could be used for in-situ corrosion monitoring against SCC-induced premature failures.

## Figures and Tables

**Table 1:** Chemical compositions of as-received SUS420J2 steel in this study, mass%.

C	Si	Mn	P	S	Ni	Cr	Fe
0.32	0.17	0.38	0.031	0.002	0.34	12.85	Bal

**Table 2:** Heat treatment procedures and mechanical properties of the two kinds of steel specimens used in this study.

Specimen #	Heat Treatment			Mechanical Properties					
	Annealing	Quenching	Tempering	*E* (GPa)	σ_0.2_ (MPa)	UTS (MPa)	EL (%)	RA (%)	HV
A	950 °C/2 h	Argon gas cooling	500 °C/20 h	218	1027	1169	12.5	45.6	367
B	950 °C/2 h		-	211	1119	1699	9.6	28.4	511

**Table 3:** Summary of the corrosion behaviors and AE results of two cases.

Specimen #	Corrosion Test	Exposure	Corrosion Behavior	AE Events
A	U-bend, 1% NaCl-droplet 99% RH, RT.	311 h	Localized corrosion	106
B		48.5 h	SCC	610
